# Evaluation of the Cobas 4800 HPV Test for Detecting High-Risk Human Papilloma-Virus in Cervical Cytology Specimens

**DOI:** 10.3390/pathogens1010030

**Published:** 2012-09-12

**Authors:** Isabella W. Martin, Heather B. Steinmetz, Claudine L. Lefferts, Larry J. Dumont, Laura J. Tafe, Gregory J. Tsongalis

**Affiliations:** 1 Department of Pathology, Dartmouth Hitchcock Medical Center and Geisel School of Medicine at Dartmouth, One Medical Center Drive, Lebanon, NH 03756, USA; E-Mails: Isabella.w.martin@hitchcock.org (I.W.M.); larry.j.dumont@hitchcock.org (L.J.D.); laura.j.tafe@hitchcock.org (L.J.T.); 2 Department of Pathology, Dartmouth Hitchcock Medical Center, One Medical Center Drive, Lebanon, NH 03756, USA; E-Mails: heather.b.steinmetz@hitchcock.org (H.B.S.); Claudine.l.lefferts@hitchcock.org (C.L.L.); 3 Department of Pathology, Dartmouth Hitchcock Medical Center, Geisel School of Medicine at Dartmouth, and Norris Cotton cancer Center, One Medical Center Drive, Lebanon, NH 03756, USA

**Keywords:** human papillomavirus, cervical cancer screening, Cobas 4800 HPV assay

## Abstract

As new platforms for high-risk strains of human papillomavirus (HR HPV) testing are introduced into the clinical laboratory, it is important to verify their performance and agreement. In this validation study, post-aliquot cervical cytopathology specimens (n = 226) were used to analyze agreement between the Invader HPV ASR assay (Hologic) and the recently FDA-approved Cobas 4800 high-risk HPV assay (Roche). Residual sample from 92 Invader positive and 134 Invader negative samples were analyzed with the Cobas 4800 test. Discordant results were further analyzed by Linear Array HPV genotype testing (Roche). To assess intra- and inter-run precision, 31 Invader positive samples were run in duplicate on the Cobas 4800 by different operators over multiple days and purchased HR HPV DNA control was run in ten replicates. Cross-contamination during cytology processing was evaluated by spiking 6 Invader negative samples with different volumes of Acrometrix HPV High Risk Positive Control and analyzed on the Cobas with 4 negative samples in between. There was significant discordance between the assays (p < 0.001; exact McNemar X^2^ test), with overall agreement of 82%. Of the 92 Invader positive samples, 58 (63%) were positive with the Cobas assay, while 34 (37%) were negative. Of the 134 Invader negative samples, 6 (4%) were positive with the Cobas while 128 (96%) were negative. The observed discordance may be attributed to the previously described false positive rate of the Invader ASR assay. The Cobas 4800 high-risk HPV assay is a viable new tool for use in the clinical setting to identify high-risk HPV.

## 1. Introduction

Cervical cancer is the second most common cause of cancer in women worldwide [1]. Over the past several decades, cervical screening has decreased the incidence of invasive cervical cancer and related mortality in the populations where it is used [2,3,4]. More recently, molecular testing has enabled detection of high-risk types of the cancer-causing human papillomavirus (HPV) in cervical specimens. Specifically, out of 15 known carcinogenic HPV strains, types 16 and 18 have been shown to be responsible for approximately 70% of cervical cancer [5]. Testing for these and other high-risk strains of HPV (HR HPV) is now an important component of cervical cancer prevention. Consensus guidelines put forth by the American Society for Colposcopy and Cervical Pathology developed at a meeting of 146 experts from 29 different professional organizations, federal agencies and national and international health organizations recommend reflex HR HPV testing for a cytologic diagnosis of atypical squamous cells of undetermined significance (ASC-US) as well as co-testing in women over 30 [6]. 

Current US Food and Drug Administration (FDA)-approved molecular methods of HR HPV screening in these specific populations include the Hybrid Capture 2 HPV DNA test (Digene Corp., Gaithersburg, MD, USA, approved in 1999), the Cervista HPV HR test (Hologic, Bedford, MA, USA, approved in March 2009), the Cervista HPV 16/18 test (Hologic, Bedford, MA, USA), the Cobas 4800 HPV test (Roche, Pleasanton, CA, USA, approved in April 2011) and the Aptima HPV assay (Gen-Probe, San Diego, CA, USA, approved in October 2011). Cervical cancer screening guidelines were written based on the performance of the Hybrid Capture 2 assay in the late 1990s and early 2000s. As new testing methods for HR HPV are approved and introduced into the clinical laboratory, it is important to verify their performance and agreement between tests and technologies. 

The Cobas 4800 high-risk HPV test is approved to detect HPV16, HPV18 and a pool of 12 other high-risk strains in pre-aliquot PreservCyt media (Hologic, Bedford, MA, USA) or Cobas PCR Cell Collection Media (Roche Molecular Systems, Inc.). The ATHENA HPV study and several smaller studies have compared the Cobas test with the Hybrid Capture 2 assay [7,8,9]. However, no studies have been published to date comparing the Cobas 4800 assay to the Invader technology. In this study, we evaluated analytical performance and interassay agreement between these two tests. 

## 2. Results and Discussion

### 2.1. Agreement

Of the 226 specimens tested by the Invader and Cobas for HR HPV, 92 were previously positive and 134 negative by the Invader assay. No specimens were excluded for inadequate cellularity/DNA content. Of the 92 Invader positive samples, 58 (63%) were positive on the Cobas 4800, while 34 (37%) were negative. Of the 134 Invader negative samples, 6 (4%) were positive by the Cobas 4800 while 128 (96%) were negative (see Table 1). In total there were 40 (18%) discordant results. This represented a significant discordance between the assays (p < 0.001; exact McNemar X^2^ test), with overall agreement of only 82%. Of the 20 randomly-selected Invader positive discordant samples analyzed by Linear Array HPV genotype testing, 4 were low-risk HPV and 12 were negative for HPV DNA. One sample lacked sufficient cellularity to obtain a result. The remaining 3 samples genotyped as HPV types of uncertain oncogenic risk (HPV types 55, 67 and IS39). 

**Table 1:** Cobas 4800 *versus* Invader high-risk strains of human papillomavirus (HR HPV) assays.

Data Summary	Cobas 4800 Positive	Cobas 4800 Negative
Invader HR Positive	58 (63%)	34 (37%)
Invader HR Negative	6 (4%)	128 (96%)

### 2.2. Precision

There was 100% concordance between the 31 positive samples run in duplicate on the Cobas 4800. The mean difference between the cycle threshold (Ct) of the two runs was 0.4 with a standard deviation of 1.4. The run-to-run coefficient of variation was 3.5%. There was not a significant difference between the runs (p = 0.9). All 10 HR HPV control samples tested positive for HR HPV with a mean Ct of 33.33 and standard deviation of 0.50 and a coefficient of variation of 1.5%. All interspersed negative samples tested negative. 

### 2.3. Cross-Contamination

Of 10 the samples re-processed on the Cytyc T-3000, the 6 spiked positive HR control samples all tested positive for HR HPV on the Cobas 4800 while the 4 interspersed negative samples all tested negative.

### 2.4. Discussion

Several studies have been published showing good agreement between the Cobas 4800 test and the Hybrid Capture 2 test (Qiagen, Gaithersburg, MD) [7,8,9]. In the present study, the comparison of the Cobas 4800 HR and Invader HPV ASR assays showed poor agreement. There was a striking difference between the agreement in the Invader positive and Invader negative groups. A high discrepancy was found in the Invader positive group with only 63% of the 92 specimens testing positive by Cobas 4800 HR analysis. This discordance may be attributed to the previously described high false positive rate of the Invader ASR assay [10]. Indeed, the majority of the 20 discordant samples selected for Linear Array analysis were, in fact, Invader false positives with 4 genotyping as low-risk HPV and 12 as HPV negative. Of the remaining 4 samples, 1 lacked sufficient cellularity to obtain a result and 3 samples genotyped as HPV types of uncertain oncogenic risk (HPV types 55, 67 and IS39). The Invader negative samples had a much higher agreement of 96% with the Cobas 4800 assay, suggesting a similar sensitivity between the two assays. In a comparison study between the FDA-approved Cervista HPV HR assay and the Digene hc2 assay, Youens *et al*., show a lower potential false positive rate with the Cervista assay [11]. This was attributed to a possible higher sensitivity of hc2 than Cervista or due to cross reactivity of the hc2 assay. A major difference between the Youens study and our current study was that the former conclusions were based on positivity rates *versus* our study which were based on testing of identical samples. These differences may reflect preanalytical differences in positivity rates of the different populations or analytical variables as described. In addition, our study compared the Cobas 4800 HR assay to the Invader HPV ASR which was validated in the laboratory as a LDT and has been in use in the laboratory for the past several years. The performance of the Invader ASR may not reflect the same performance of the FDA-approved Cervista HR HPV assay.

As noted by Kinney *et al*., the false-positive rate of any HPV screening assay is problematic as it can lead to over-referral to colposcopy and over-treatment causing both psychological stress for the women involved and inefficient use of health care resources [10]. The Cobas 4800 assay may offer improved specificity without sacrificing sensitivity in detecting HR HPV. 

We recognize the small sample size as a limitation of this study and the fact that the Cobas testing was performed on post-aliquot residual liquid cytology samples. Additionally, only 20 of the 40 discordant results were genotyped by the Linear Array test. However, the fact that the vast majority of these 20 discordant samples randomly chosen for Linear Array testing indicated false positive Invader results supports the above hypothesis. The possibility exists that discordance is not solely due to false positive Invader results and could be due to sampling error. It is also possible that the Cobas 4800 test produced false negative results. However, the detection of human beta-globin DNA helps to reduces false negatives by serving as an internal control of adequate sample cellularity and extraction.

The FDA approved the Cobas 4800 HPV assay in the setting of pre-aliquot samples, those that have not been processed for cytology by an instrument such as the Cytyc T-3000. However, obtaining “pre-aliquot” samples for HR HPV testing presents logistical barriers for many laboratories, due in part to the “reflex” nature of a large part of HPV testing. Because the PreservCyt specimens are first and foremost cytology specimens and subsequent HPV testing is often a “reflex” test performed only after a cytologic diagnosis of ASC-US has been rendered, obtaining pre-aliquot specimens for HPV testing is impractical and could compromise the cytology exam. We observed no detectable carryover contamination from cytology processing with the Cytyc T-3000 when samples were subsequently analyzed on the Cobas 4800 and HPV detection rates were as expected. 

The Cobas 4800 achieved excellent precision with a mean run-to-run difference in cycle threshold of 0.4 and a run-to-run coefficient of variation of 3.5%. In general clinical testing, a coefficient of variation of less than 5% is considered desirable. Precision in testing is an important quality for any laboratory test as it indicates that the chance of random variation in test results is small.

## 3. Experimental Section

### 3.1. Hologic Invader HPV ASR Assay

Our laboratory validated the Invader HPV ASR for clinical use in screening Surepath and PreservCyt liquid cytology samples before the assay was FDA approved as the Cervista HR HPV assay. The test uses the Invader technology to qualitatively detect HR HPV DNA from a pool of 14 HR HPV strains (16, 18, 31, 33, 35, 39, 45, 51, 52, 56, 58, 59, 66, 68). The assay requires a minimum volume of 2 mL of PreservCyt liquid cytology specimen and incorporates a Tecan EVO 150 robotic workstation using sample DNA extracted with the Agencourt Genfind magnetic bead separation method. After extraction, the HPV-specific reagents are then presented in three probe pools that correspond to four HPV virus groups based on phylogenetic relatedness. Additionally, a human DNA reagent specific for the human histone 2 gene (HIST2H2BE) serves as an internal control assuring the sample contains sufficient cellularity/DNA and that no inhibitory substances are present. If the internal control is negative, results are reported as insufficient specimen quality or quantity. In this study, all samples were analyzed according to the laboratory developed and validated protocol using the ASR.

### 3.2. Roche Cobas 4800 HPV Assay

The Cobas 4800 HPV assay is a qualitative test for the presence of HPV 16, 18 and a pool of 12 other HR HPV types 31, 33, 35, 39, 45, 51, 52, 56, 58, 59, 66 and 68. This assay requires 1 mL of liquid cytology sample. DNA is extracted, purified and prepared for PCR from PreservCyt cytology specimens by the Cobas x480 instrument which is integrated into the Cobas 4800 platform. Target DNA is then amplified by PCR with subsequent nucleic acid hybridization on the Cobas z480 analyzer. The Cobas 4800 assay simultaneously tests for human beta-globin DNA as an internal control of sufficient specimen cellularity. In addition, a positive and negative control specimen is included in each run. In this study, the Cobas 4800 HPV test was performed according to the recommendations of the manufacturer with the exception of using post-aliquot samples. 

### 3.3. Linear Array HPV Genotyping Test

The Linear Array HPV Genotyping Test (Roche Molecular Diagnostics, Inc) was used to amplify target DNA by PCR and hybridize nucleic acids with probes designed to detect thirty-six HPV genotypes and one HPV 82 variant [6, 11, 16, 18, 26, 31, 33, 35, 39, 40, 42, 45, 51, 52, 53, 54, 55, 56, 58, 59, 61, 62, 64, 66, 67, 68, 69, 70, 71, 72, 73 (MM9), 81, 82 (MM4), 83 (MM7), 84 (MM8), IS39, and CP6108] [12]. The assay also contains an internal control in the form of the human β-globin gene. The HPV Linear Array test was performed according to the manufacturer’s recommendation with the following exception: DNA extracted on the Tecan EVO150 with the Agencourt Genfind reagents.

### 3.4. Sample Selection

At the time of this study, our laboratory performed reflex testing for HR HPV using the Invader HPV ASR assay on specimens with a cytologic diagnosis of ASC-US as well as co-testing for women over the age of 30 years upon clinician request. To perform this HR HPV testing, 4 mL of residual (“post-aliquot”) cytology specimen in PreservCyt medium were aliquotted from the original collection vial and 1 mL of this sample was then used in the assay. Before HR HPV testing, these samples had been previously processed with the Cytyc T-3000 (Hologic, Bedford, MA), an automated slide preparation technology for liquid-based cytology. In this study, we identified consecutive cervical cytopathology specimens (n = 226) between October 2011 and December 2011 that had already undergone Invader HPV testing and that had adequate residual volume in the PreservCyt medium for additional testing. 

### 3.5. Agreement

Each of the residual PreservCyt cytology specimens was analyzed with the Cobas 4800 assay within three months of initial collection. PreservCyt specimens were stored at 4 °C. Twenty Invader-positive discordant samples were randomly selected and analyzed using the Roche HPV Linear Array to detect the presence or absence of HPV DNA and to genotype positive samples. 

### 3.6. Precision

To assess precision, 31 Invader positive samples were run in duplicate on the Cobas 4800 by different operators over multiple days. Additionally, HPV High Risk control material was purchased from Acrometrix, Inc and was analyzed on the Cobas 4800 in 10 replicates within the same run with previously tested negative samples between each positive control.

### 3.7. Cross-Contamination

To control for possible sample-to-sample cross-contamination during cytology processing by the Cytyc T-3000, 6 Invader negative samples were spiked with different volumes of Acrometrix HPV High Risk Positive Control: 2 samples with 4 mL, 2 with 2 mL and 2 with 1 mL. These 6 spiked samples were re-processed on the Cytyc T-3000 with 4 previously negative samples interspersed between the controls in the following order: 4 mL, blank, 2 mL, 1 mL, blank, 4 mL, blank, 2 mL, 1 mL, blank. Each sample was analyzed for HR HPV on the Cobas 4800.

## 4. Conclusions

In summary, poor agreement between the Cobas 4800 and Invader HPV ASR assays can be attributed to the false positive rate of the Invader assay as determined by HPV genotyping. We conclude that the Cobas 4800 assay offers an important advantage in HR HPV sensitivity on a platform with excellent precision. The Cobas 4800 also offers other advantages such as simultaneous genotyping of HR HPV types 16 and 18, lower false positive rate than the Invader assay, smaller batch size (between 24 and 96 samples) and more rapid turn-around time (5 hours), thus providing a viable new tool for use in the clinical setting to identify HR HPV. 
